# Effect of Tamarind Gum on the Properties of Phase-Separated Poly(vinyl alcohol) Films

**DOI:** 10.3390/polym14142793

**Published:** 2022-07-08

**Authors:** Madhusmita Rawooth, SK Habibullah, Dilshad Qureshi, Deepti Bharti, Ankit Pal, Biswaranjan Mohanty, Maciej Jarzębski, Wojciech Smułek, Kunal Pal

**Affiliations:** 1 Department of Biotechnology and Medical Engineering, National Institute of Technology, Rourkela 769008, Odisha, India; madhu.rawooth@gmail.com (M.R.); dilshadq786@gmail.com (D.Q.); deeptibharti94@gmail.com (D.B.); 2 Department of Pharmaceutics, Institute of Pharmacy and Technology, Salipur, Cuttack 754202, Odisha, India; skhabibullah.lucky@gmail.com (S.H.); www.ankit1507@gmail.com (A.P.); 3 Department of Physics and Biophysics, Faculty of Food Science and Nutrition, Poznań University of Life Sciences, Wojska Polskiego 38/42, 60-637 Poznan, Poland; 4 Institute of Chemical Technology and Engineering, Poznan University of Technology, Berdychowo 4, 60-695 Poznan, Poland; wojciech.smulek@put.poznan.pl

**Keywords:** poly(vinyl alcohol), phase separation, tamarind gum, composite films, solvent casting, drug release

## Abstract

The current study aims to evaluate the effect of tamarind gum (TG) on the optical, mechanical, and drug release potential of poly(vinyl alcohol) (PVA)-based films. This involves preparing PVA-TG composite films with different concentrations of TG through a simple solvent casting method. The addition of TG has enhanced the phase separation and aggregation of PVA within the films, and it becomes greater with the increase in TG concentration. Brightfield and polarized light micrographs have revealed that aggregation is favored by forming crystalline domains at the PVA-TG interface. The interconnected network of PVA-TG aggregates influenced the swelling and drying properties of the films. Using Peleg’s analysis, the mechanical behavior of films was determined by their stress relaxation profiles. The addition of TG has made no significant changes to the firmness and viscoelastic properties of films. However, long-durational relaxation times indicated that the interconnected network might break down in films with higher TG concentration, suggesting their brittleness. The controlled release of ciprofloxacin in HCl solution (0.5% (*w*/*v*)) appears to decrease with the increase in TG concentration. In fact, TG has inversely affected the impedance and altered the ionic conductivity within the films. This seems to have directly influenced the drug release from the films as the mechanism was found to be non-Fickian diffusion (based on Korsmeyer–Peepas and Peppas–Sahlin kinetic models). The antimicrobial study using *Escherichia coli* was carried out to evaluate the activity of the drug-loaded films. The study proves that TG can modulate the properties of PVA films and has the potential to fine-tune the controlled release of drugs from composite films.

## 1. Introduction

Controlled delivery of drugs has recently emerged as an intriguing research area [1,2,3,4]. In recent decades, many efforts have been expended to develop a unique drug release system that can control and prolong the release time and improve the drug’s bioavailability, efficacy, and safety [5]. In a controlled manner, an effective delivery system should transport the anticipated drugs to the targeted sites and release the drug [6]. A controlled release system can be prepared by incorporating drugs within a substrate, generally constituted of polymeric or polymeric composite-based materials, with specific chemical, physical, biological, electrical, and mechanical properties [7,8]. 

Poly(vinyl alcohol) (PVA) is a synthetic polymer that is derived from the hydrolysis of polyvinyl acetate. It is a semi-crystalline polymer. Commercially available PVA can be divided into fully and partially hydrolyzed PVA based on the extent of hydrolysis [9]. PVA is soluble in hot water [10] and exhibits a high swelling in aqueous solutions [11]. The wide use of PVA in various fields is due to its unique properties, such as its simple chemical structure, non-toxicity, bio-adhesiveness, biocompatibility, inertness, and elasticity. Hence, PVA is widely used for biomedical applications. Furthermore, PVA, in many cases, is unified with natural or synthetic polysaccharides to achieve desirable characteristics for a specific application [12]. The polymer matrices of PVA have been widely used to design wound dressings [12], artificial organs [13], and drug delivery systems [14,15,16]. PVA-based hydrogel films exhibit great porosity, drug-loading capacity, and improved mechanical properties, allowing easy handling, good skin adherence, and enhanced transdermal drug delivery efficiency [17]. The electrospinning technique has been successfully tested to create nanofibrous membranes of poly(vinyl alcohol) (PVA) and lysine (Lys). Such films have shown sufficient properties to be explored as drug delivery systems for wound dressing applications [18]. The composite films of PVA, chitosan and gelatin loaded with honey were evaluated as wound dressings [19]. The hydrogels were found to promote wound healing in rats. The chitosan/PVA hydrogels also have antimicrobial properties [20]. Macroporous composites of PVA and graphene oxide nanocomposite films have been examined as transdermal drug delivery systems [21]. Including graphene oxide within the PVA matrices allowed the researchers to manipulate the drug delivery characteristics from the composite matrices. The nanocomposite membranes of PVA and cellulose nanocrystals have been successfully explored for the delivery of curcumin to treat liver and breast cancer [22].

Similar to PVA, there are various naturally occurring polysaccharides, e.g., starch, pectin, alginates, chitosan, gellan, xanthan gum, and tamarind gum. They have also been widely used to develop drug delivery systems in the last few decades. This has been attributed to the beneficial properties exhibited by the polysaccharides from natural sources [23]. Some of the crucial properties of polysaccharides include biodegradability, biocompatibility, and bio-adhesiveness [24,25]. Using these natural polymers is popular due to their availability and cost effectiveness. Tamarind gum (TG), a promising non-ionic plant-derived natural polysaccharide, is collected from the endosperm of the seed of Tamarindus indica (Family: Leguminosae). Chemically, TG is galactoxyloglucan and has been reported to have a typical molecular weight of 52,350 Daltons [26]. However, its properties may vary depending on the molecule’s weight range, a typical phenomenon for different polysaccharides [27]. It contains glucose, xylose, and galactose [28,29]. Despite being insoluble in organic solvents, TG disperses and hydrates readily in warm water to form viscous gels [30] and stabilizes emulsion systems [31]. The primary constituent of the powdered TG is xyloglucan, which contributes significantly to the viscosity property of the aqueous solutions and the formation of hydrogels [32]. Chemical modification of TG can be carried out by carboxymethylation, acetylation, thiolation, amination, and hydroxyl alkylation. These modifications can alter its swelling, viscosity, degradability, and hydration properties [26]. TG is widely considered a useful food additive, especially as a plant-derived substitute for animal gelatine [33]. Nevertheless, TG has been extensively used to develop pharmaceutical applications. Adding TG powder to the orally disintegrating tablets can enhance its mucoadhesion and promote disintegration [34]. The composite microspheres of TG and alginates, prepared by the ionotropic gelation method, have been used for the sustained release of dalfampridine [35]. The emulsifying properties of TG have been explored to develop pharmaceutical emulsions with improved stability [31]. In a recent study, carbon nanotubes loaded with gelatin–TG hydrogels were proposed as drug delivery matrices for wound healing, tissue repair, and regeneration [36]. In our present investigation, we developed PVA–Tamarind gum composite films containing different concentrations of TG. The objective of the current work is to analyze the role of TG on the physicochemical properties of PVA film.

## 2. Results and Discussion

### 2.1. Visual Appearance of Composite Films

**Figure 1:**
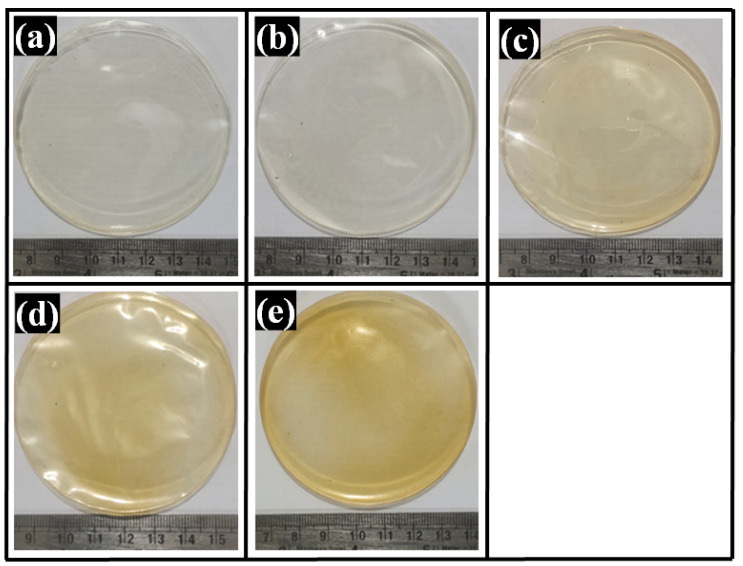
Pictures of the films: (**a**) F0, (**b**) F1, (**c**) F2, (**d**) F3, and (**e**) F4.

The composite films were made by solvent casting, one of the oldest and simplest techniques [37]. GTA was used as a crosslinking agent to improve the handling properties of the films. This can be reasoned to reduced mobility of the polymer chains due to the crosslinking reaction, which increased the films’ tensile strength [38]. The physical and mechanical properties of the PVA/ cellulose film were significantly improved upon GTA addition [39]. The composite films were light yellowish in color and transparent (Figure 1). All the films were found to be smooth and flexible. The shades of yellow were observed to be increased as TG content was increased from F0 to F4. An increased TG concentration reduced the apparent transparency of the composite films. Thus, the convenient, straightforward solvent casting method could prepare the PVA composite films with 1% to 10% TG. 

### 2.2. Microscopic Analysis

**Figure 2:**
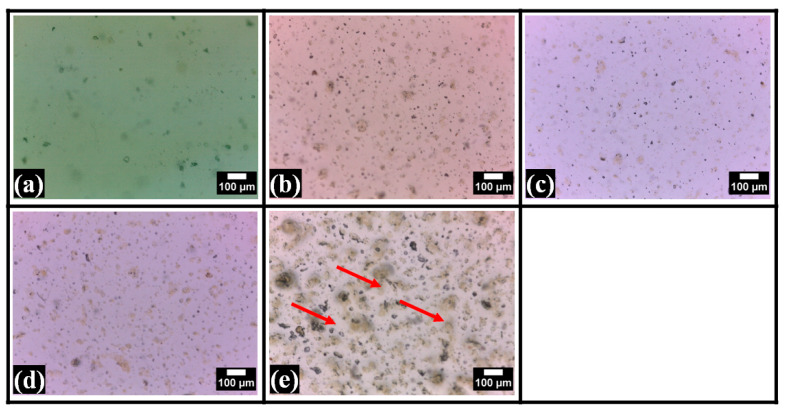
Bright-field micrographs of the films: (**a**) F0, (**b**) F1, (**c**) F2, (**d**) F3, and (**e**) F4.

The control film, which contained no TG, appeared relatively smooth compared to the TG-containing films. Some particulate structures were seen in F0. These particulate structures can be related to the crystalline regions of the PVA matrix, which is conventionally regarded as semi-crystalline [40]. The brightfield micrographs of the TG-containing films revealed dispersions of irregular- and globular-shaped structures throughout the continuum polymeric matrices (Figure 2). TG is hydrophobic in nature. The increased concentration of TG in composite films led to the formation of more phase-separated structures, which were globular, as evident by the micrographs [41]. Numerous particulate structures, along with some large-sized globular structures, were present in F1. With a further increase in TG in F2 and F3, the larger particles became smaller, but the smaller particulate structures increased. Interestingly, in F4, many more prominent irregular-shaped structures were observed throughout the matrix (marked in red). The results indicated that the matrix of F2 was relatively more homogenous than the other TG-containing films. The existence of a dispersed phase in all TG-containing films stated the formation of the phase-separated films. The appearance of this type of polymeric architecture has been reported in [42], where the authors have synthesized phase-separated composite films of PVA and carboxymethyl TG. In the synthesized films, the polysaccharide phase was embedded within an unceasing polymer matrix of PVA. This was also seen in our study. At higher concentrations of carboxymethyl TG, the films revealed an interconnected polysaccharide network. However, in our case, no interconnected network of the dispersed phase was observed in any of the films.

**Figure 3:**
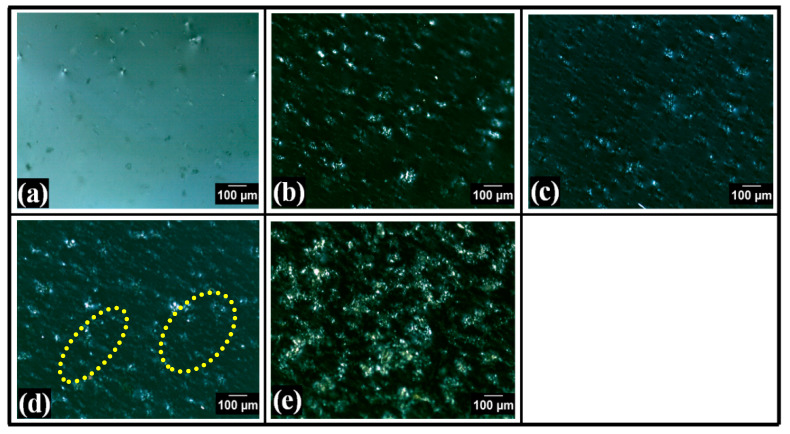
Polarized micrographs of the films: (**a**) F0, (**b**) F1, (**c**) F2, (**d**) F3, and (**e**) F4.

Subsequently, the films were observed under the polarized light microscope. The polarized micrographs (Figure 3) also supported the observation of brightfield micrographs. It was found that some crystalline regions appeared as the bright regions within the polymer matrix of F0. This confirms the semi-crystalline nature of the PVA film that was estimated from the brightfield micrographs. The overall brightness of F0 appeared to be higher than the TG-containing films. The TG-containing films showed dispersed phases throughout the polymer matrices and concordance with the brightfield microscopy. Interestingly, it was also seen that the crystalline region was mainly present over the globular structures of the polysaccharide phase. This indicates that the crystalline domains (brighter regions) of PVA were mainly formed at the interface of the PVA and TG phases [43]. It can be confirmed by adding a surfactant to the formulations in our future study. Furthermore, in F4, it was observed that the dispersed phase structures were interconnected (marked in yellow), which is in concurrence with the observations made in the films of PVA and carboxymethylated TG [42]. In their results, the SEM study revealed the globular structure of carboxylated TG within the PVA matrix. The visual analysis of the films suggested that the crystalline domains in F2 were the least among all the films, which was followed by the crystalline domains in F3, F1, and F5, respectively. In [35], it has been reported that the crystallinity of the polymeric matrices is dependent on the hydrogen bonding within the film components. Hence, hydrogen bonding in the films would be in the order of F0 >> F4 > F1 > F3 > F2. The crystalline regions behave as crosslinking points, which may affect the swelling properties of the films. Since an increase in the crosslinking density decreases the swelling ratio of the polymeric matrices, the extent of swelling may occur in the order of F2 > F3 > F1 > F4 > F0.

In their work, Yadav et al. [44] used nanotubes and graphene oxide to fill PVA and carboxymethyl tamarind gum and found that the resulting films were homogeneous. Their microscopic SEM observations indicated the presence of globular structures placed within an uninterrupted polymer matrix were present. Another study using PVA and tamarind gum was realized by Qureshi et al. [45]. Even when the dispersion provides relative homogeneity, the composites filled with bentonite exhibit lower transparency than non-filled films. They also possessed globular structures visible in microscopic analyses, similar to our study.

### 2.3. Swelling Study

**Figure 4:**
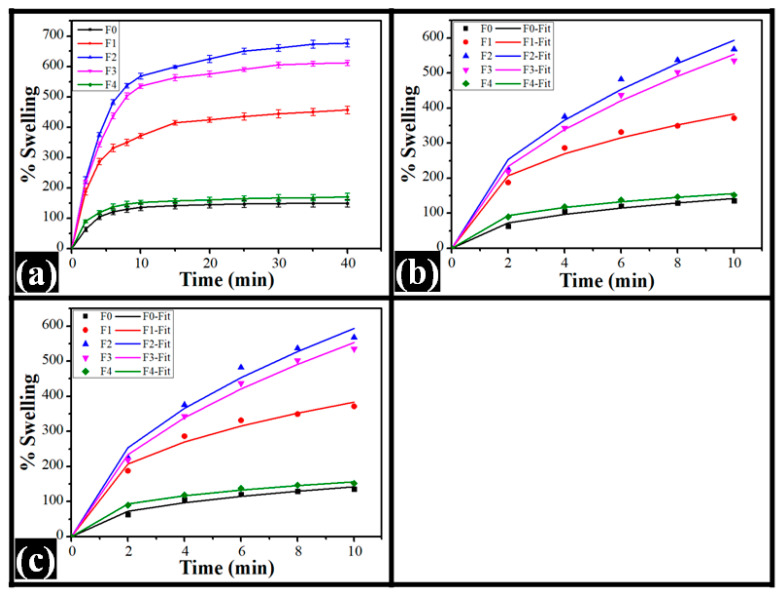
Swelling analysis: (**a**) Representation of %swelling profiles of the films, model fitting (**b**) Korsmeyer–Peppas, and (**c**) Peppas–Sahlin.

The results of the swelling study of the composite films are compiled in Figure 4. The swelling study was examined using double-distilled water (pH 6.8), and all the films quickly absorbed water during the first 10 min of the swelling study. The TG films showed higher swelling characteristics than the control film (F0). TG-containing films showed a composition-dependent swelling behavior. At the end of the experiment (post 40 min), the %swelling of F0 was 149.57 ± 12.46%. After that, in F1, where TG was present in the lowest concentration, the %swelling was significantly increased to 457.10 ± 12.60%. With the further increase in TG content of F2, the %swelling at the end of 40 min was consequently increased to 676.86 ± 12.67%. Interestingly, with the further increase in the TG content in F3 and F4, the %swelling was reduced to 611.82 ± 8.69% and 170.50 ± 11.41%, respectively. The increase in the %swelling of the films can be explained by the expansion of the three-dimensional hydrophilic polymer network when the films come into contact with water. The %swelling of the composite films with higher TG content was lower than those with lower TG content. A high TG supports more hydrogen bonding, further assisting in the efficient crosslinking of the PVA matrix. Thus, higher crosslinking will result in reduced swelling. The FTIR study confirms the decrease in hydrogen bonding from F0 to F2 [46]. The swelling behavior of the films followed the same order as predicted from the analysis of the polarized micrographs. This suggests that the formation of the crystalline regions within the PVA matrices affected the swelling kinetics. In general, a higher number of crystalline regions hampered the swelling kinetics of the films.

The mechanics of water penetration into the polymer matrix were estimated by fitting the %swelling profiles to the Korsmeyer–Peppas mathematical model (Equation (1) [47]. The initial 10 min of the experimental data was used for the modeling. The mathematical parameters obtained from modeling the experimental data are represented in Table 1. The “k” value, which suggests the diffusion of the water molecules within the polymer matrices of the control film (F0), was the lowest. The “k” values of the TG-containing films were higher than the control (*p* < 0.05). When the TG content was on the lower side, an increase in the TG content improved the “k” value. In other words, the “k” value of F1 and F2 increased with the TG content increase. However, the change in the “k” value between F1 and F2 was statistically insignificant (*p* > 0.05). A further increased TG content decreased the “k” value in F3 and F4, respectively. The “k” values of F1 and F3 were statistically insignificant (*p* > 0.05). This suggested that when the TG content was low (i.e., in F1 and F2), the diffusion of the water molecules within the polymer network was promoted compared to the control film. However, increased TG content in F3 and F4 caused a hindrance in the diffusion of the water molecules within the polymer network more significantly than F2, which revealed the highest %swelling. This indicates that at higher concentrations of TG, there must be changes in the inter/intramolecular interaction mechanics, which significantly alters the films’ swelling characteristics. The TG-containing diffusion exponent (n) was observed to be lower than F0. The “n” value of all the films (F0–F4) was greater than 0.45, indicating that the water transport within the films followed a non-Fickian diffusion transport phenomenon. Again, the “n” value of the TG-containing films was statistically insignificant (*p* > 0.05) compared to the control film (F0).

Thereafter, Peppas–Sahlin (PS) Equation (2) model was used for fitting the obtained swelling profiles [48]. Herein, similar to the Korsmeyer–Peppas mathematical model, the initial 10 min of experimental data was used for the modeling. The polymer relaxation constant (k_r_) values and “k” values (from the Korsmeyer–Peppas mathematical model) of all the films were the same. This is possible only if the polymer relaxation process was the sole cause for the swelling of the films. The “m” value of all TG-containing films was statistically insignificant (*p* > 0.05) compared to the control film.

(1) $$F=\left(\frac{M_{t}}{M}\right)=k\cdot t^{n}$$

where $\frac{M_{t}}{M}$ is the absorbed water fraction in the polymeric matrix at the time “t”, “k” signifies the release rate constant, and “n” is the diffusion exponent.

(2) $$F=\left(\frac{M_{t}}{M_{0}}\right)=k_{d}\cdot t^{m}+k_{r}\cdot t^{2m}$$

where $\frac{M_{t}}{M_{0}}$ is the water fraction absorbed in the matrix at the time “t”; “k_d_” is for diffusion occurring due to Fickian release; “k_r_” is the diffusion resulting from polymer relaxation; and “m” is the diffusion exponent.

The swelling properties of tamarind gum-based films were the subject of several studies. The work of Qureshi et al. [45] contains the investigations of bentonite-filled PVA–tamarind gum composites. The authors observed that the swelling properties (tested in SBF at pH 7.4) of the prepared bentonite-containing films were significantly higher than the control, accounting for the bentonite’s water holding capacity. Additionally, Mali et al. [49] noticed that some optimum carboxymethyl tamarind gum concentration provides the highest swelling properties. The authors explained this phenomenon with a decrease in the films’ crosslinking densities. Moreover, they suggested that at an optimum concentration of carboxymethyl tamarind gum, this hydrogel network structure can expand to absorb a large amount of medium. This was attributed to the deprotonation of carboxylic acid groups and the consequent increase in repulsive forces among the negatively charged carboxylate ions. 

### 2.4. Transparency Study

**Figure 5:**
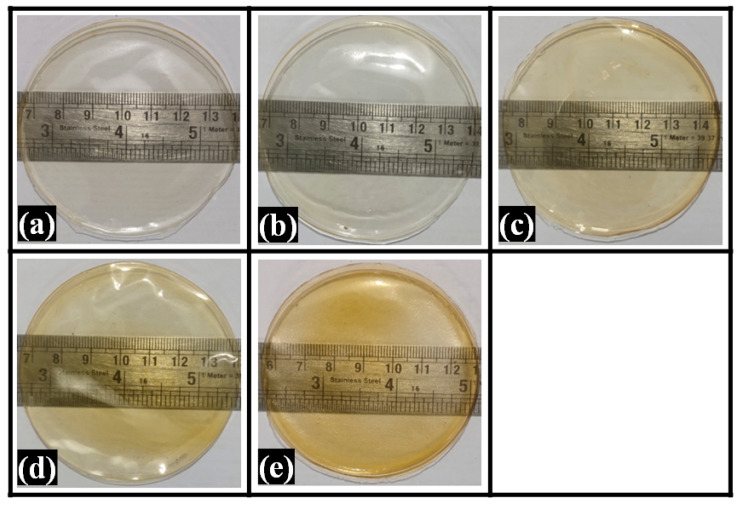
Digital images of the composite films (**a**) F0, (**b**) F1, (**c**) F2, (**d**) F3, and (**e**) F4.

Visual inspection for transparency of films was carried out by reading the numbers on a ruler that was kept under the films (Figure 5). The numbers on the ruler were easily read in all films. The films acquired an increase in yellowish tinge with the rise of TG content. Further, the transparency of the films was then analyzed by UV-visible spectroscopy. Figure 5 represents UV-visible transmittance spectra in the wavelength range of 280 nm to 900 nm. The control film (F0) showed a high UV-visible transmittance. The wavelength region of 280 nm to 320 nm, 320 nm to 400 nm, and 400 nm to 700 nm is considered UVB, UVA, and visible range, respectively [50]. The average %transmittance of pure PVA film was 52.57 ± 0.62% in the UVB region. The average %transparency of the films in the UVB decreased gradually upon increasing the TG content. The average %transparency of the TG-containing films was 47.29 ± 0.46%, 41.93 ± 0.54%, 36.75 ± 0.36%, and 33.09 ± 0.29% for F1, F2, F3, and F4, respectively.

A significant change in the average %transparency of both control and TG-containing films was also noticed in the UVA region. The average %transmittance of the control film was recorded as 78.40 ± 0.38%, which was higher than the average %transparency of the film in the UVB region. Likewise, the average %transparency of TG-containing films was also increased to 64.69 ± 0.38% (F1), 56.85 ± 0.31% (F2), 53.39 ± 0.36% (F3), and 47.37 ± 0.37% (F4). It is to be noted that UV radiation having a wavelength between 290 nm and 350 nm is most harmful to the human body [51]. The result suggests that the inclusion of TG within the films improved the UV-shielding effect in a concentration-dependent manner. This observation could be expounded on the absorption of the UV radiation by the TG, a polysaccharide. A similar finding of the lessening of transparency within the UV region by a polysaccharide has been reported in hydroxypropyl methylcellulose/hydroxypropyl starch-based phase-separated films [52]. In [53], a reduction in UV region transparency of HPMC/pectin films was observed with the upsurge in pectin (a polysaccharide) content. Furthermore, increased TG content reduced the film’s transparency due to the increased TG density within the films, which corresponds with previously cited studies of Yadav et al. [44] and Qureshi et al. [45]. This was explained by decreased interchain polymer spacing as the TG content is increased [54].

**Figure 6:**
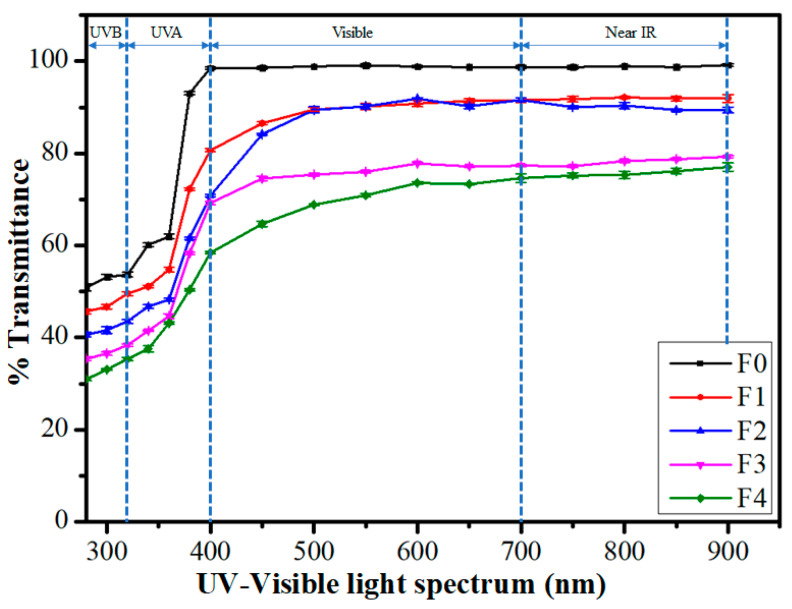
Transmittance spectra of the composite films.

The transmittance spectra of the control film in the visible range (400 nm–700 nm) did not show any significant changes (Figure 6). The average %transparency of F0 was 98.91 ± 0.23% in the visible range. The TG-containing films exhibited lower average %transparency compared to the control film. F1 and F2 did not differ significantly in the average transparency in the visible range. The average %transparency of F1 and F2 was 90.00 ± 0.50% and 89.59 ± 0.43%, respectively. However, the visible spectra of F1 and F2 in the wavelength region of 500 nm and 700 nm were similar. The average %transparency of F3 and F4 was reduced with an increased TG content. The average %transparency of films F0 and F1 was higher than 90% from 400 nm to 700 nm, which is considered an ideal parameter for hydrogel films in biomedical applications [55].

Similar to the visible region, the average %transparency of the films in the near infrared region (NIR) (700–900 nm) was 98.91 ± 0.23%, 91.95 ± 0.54, 89.82 ± 0.50, 78.38 ± 0.26, and 75.92 ± 0.73 for F0, F1, F2, F3, and F4, respectively.

### 2.5. FTIR Analysis

**Figure 7:**
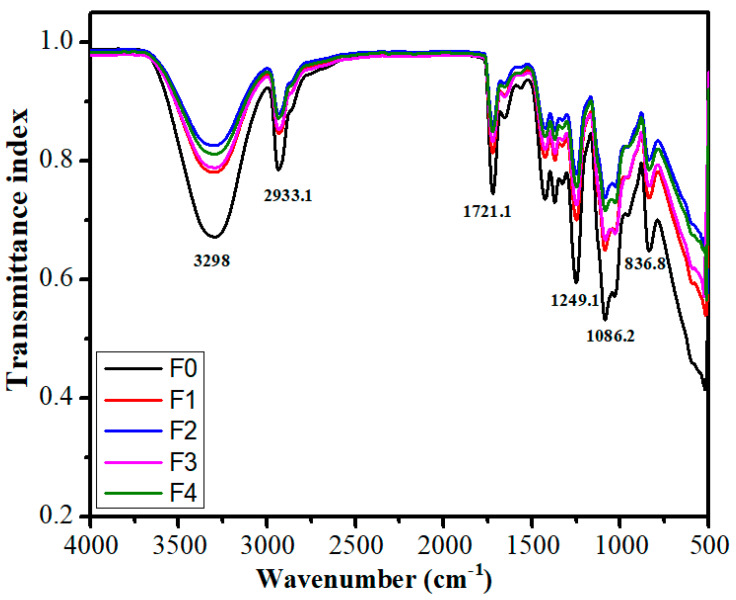
FTIR spectra of composite films.

The FTIR analysis was performed to assess the PVA, glutaraldehyde, and TG interactions within the composite films. FTIR spectra of the control film (F0) and TG-containing films (F1–F4) are presented in Figure 7, and the important peaks are tabulated in Table 2. The FTIR spectrum of F0 showed broadband between the wavenumber range of 3700 cm^−1^ and 2997 cm^−1^. This broadband at the wavenumber of 3298 cm^−1^. This peak can be related to hydrogen bonding and -OH stretching vibration [56]. A higher hydrogen bonding is expected to result in increased crystallinity of the PVA matrices. In addition, different signals were observed at 2933.1 cm^−1^, 1721.1 cm^−1^, 1655.1 cm^−1^, 1564.4 cm^−1^, 1424.3 cm^−1^, 1370.7 cm^−1^, 1327.4 cm^−1^, 1249.1 cm^−1^, 1086.2 cm^−1^, 1032.6 cm^−1^, 958.4 cm^−1^, and 836.8 cm^−1^. The signal at 2933.1 cm^−1^ corresponded to the stretching of the alkyl (C-H) group of PVA [57]. The signals positioned at 1564.4 cm^−1^ and 1424.3 cm^−1^ ascribed to bending vibration of -CH_2_. The bands at 1370.7 cm^−1^ and 1327.4 cm^−1^ were for C-H bending of -CH_3_ groups of PVA [58,59,60,61,62,63]. The signal at 1721.1 cm^−1^ corresponds to the carbonyl (C=O) group stretching vibration, which could be present in molecules of some non-saccharides components of tamarind gum-like proteins or carboxylic acids [58,59]. The signal at 1655.1 cm^−1^ can be attributed to the stretching vibration of C=C. The peak at 1249.1 cm^−1^ could be attributed to the formation of a C-O-C bond due to the reaction of -OH of PVA and -CHO of GTA [60]. The signals recorded at 1086.2 cm^−1^ and 1032.6 cm^−1^ can be assigned to C-O stretching of the groups present in the crosslinked PVA films, while the signal at 958.4 cm^−1^ and 836.8 cm^−1^ could be attributed to alkyl (C-H) bending and rocking of PVA molecule respectively [64].

The FTIR spectra of all TG-containing films were also studied. The spectra analysis confirmed the presence of all the signals of F0 in the TG-containing films. The increased content of TG in the composite films did not show any significant changes in the signal position except in F2 and F4. T-OH stretching vibration signal at 3298 cm^−1^ of F0 shifted to a higher wavenumber by 4.1 cm^−1^ (3302.1 cm^−1^) in F2. Compared to F0, the broadband of F2 appeared shallow in the 3700 cm^−1^ and 2997 cm^−1^. The peaks recorded at 1564.4 cm^−1^ in F0 blue-shifted to 1576.8 cm^−1^ in F4. The shift to a higher wavenumber by 12.4 cm^−1^ was observed for the signal. This suggested that the bending vibration of CH2 occurred at higher energy, which can be associated with a higher amount of protein present in TG in F4 [65].

**Figure 8:**
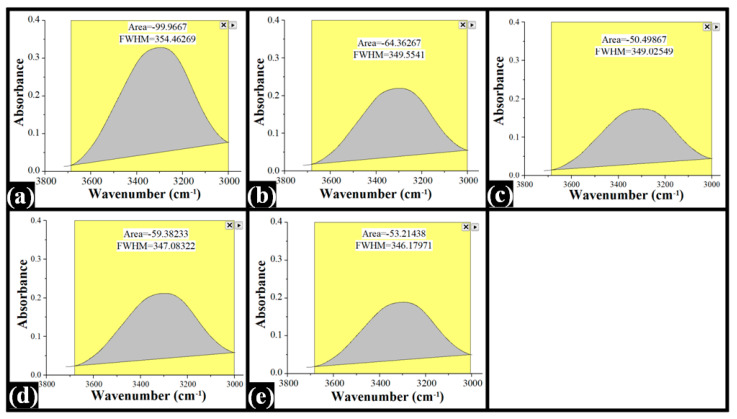
FTIR spectra of composite films (AUP). (**a**) F0; (**b**) F1; (**c**) F2; (**d**) F3; (**e**) F4.

The area under the peak (AUP), a marker of hydrogen bonding, of all the TG-containing films and the control films was calculated (Figure 8). The AUP of the TG-containing film was significantly lower than the control film. The AUP of the control film was observed as 99.97, whereas AUPs of the TG-containing films were 64.36, 50.50, 59.38, and 53.21 for F1, F2, F3, and F4, respectively. In general, the addition of TG reduced the hydrogen bonding inside the composite films. Interestingly, the order of crystallinity observed from the microscopy and estimated from AUP was similar, except for F4.

The crystallinity of a polymer matrix can affect its swelling capability. Accordingly, the analysis of the correlation between AUP and swelling becomes necessary. As mentioned earlier, there was a marked reduction in the AUP values in F1 and F2, respectively, with TG in the PVA matrices. In fact, the AUP of F2 was the lowest. This can explain the highest %swelling in F2. Since the extent of hydrogen bonding was lowest in F2, this resulted in reduced physical crosslink points and promoted the water molecules’ admission within the F2 matrix. An additional increase in TG content in F3 increased the AUP, followed by a decrease in the AUP in F4. The AUP value of F3 was in between the AUP values of F1 and F4. The %swelling of F3 was also in between the %swelling of F1 and F4. Interestingly, the AUP value of F4 was slightly higher than F2. However, its %swelling was nearly equal to F0, the lowest among all the films. This observation was quite confusing. Such a perverse observation can be clarified by the increased hydrophobic interactions within the TG phase, which was highest in F4. The increased hydrophobic interactions may have significantly barred the inflow of the water molecules within the matrix of F4. 

### 2.6. Stress Relaxation Study

**Figure 9:**
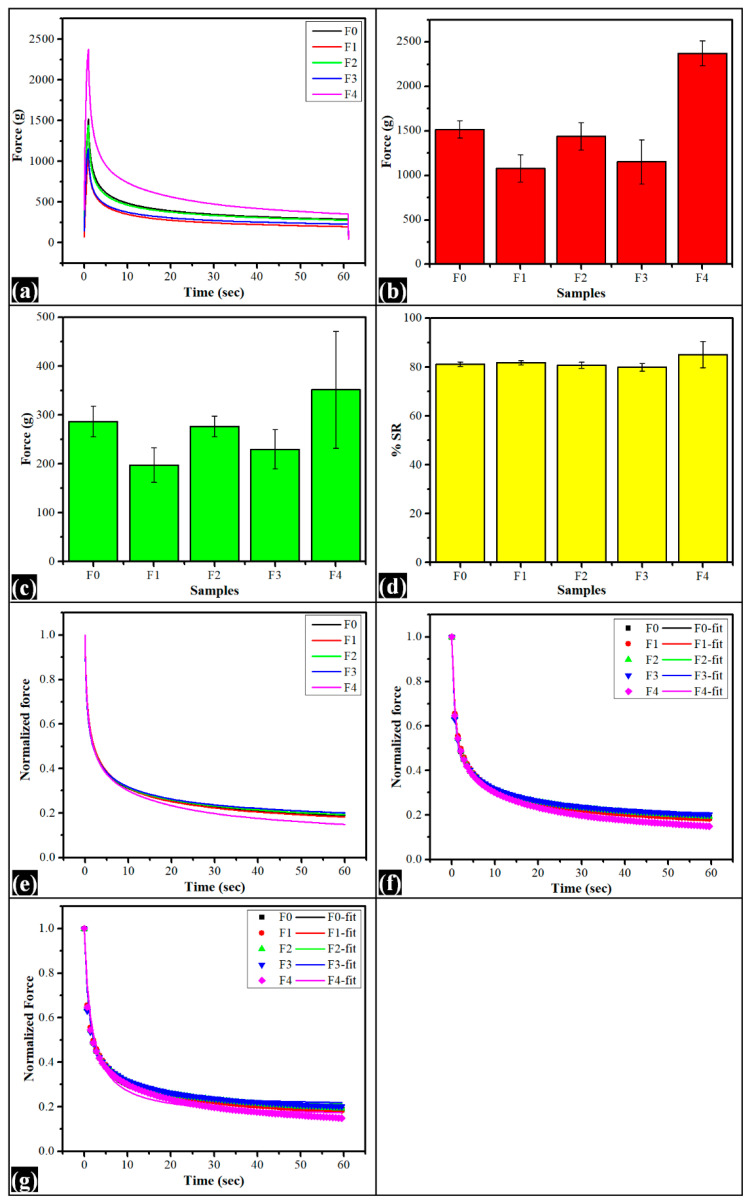
Mechanical parameters of the films: (**a**) Stress relaxation profiles, (**b**) F_max_, (**c**) F_min_, (**d**) %SR profile, (**e**) normalized stress relaxation profiles, (**f**) Wiechert modeling, (**g**) Peleg’s model fitting. The data are represented as average ± standard deviation of triplicate.

The mechanical behavior of the control film (F0) and the TG-containing composite films were assessed through a stress-relaxation (SR) study. The mechanical properties of the polymer blends can be correlated with the microstructures, system’s homogeneity, crystallinity, and polymeric interaction [66]. The SR study was performed in the extension mode. Figure 9a–g shows the viscoelastic profiles and tabularized in Table 3. The probe sensed the force value during the stretching of the film, which reached its maximum value (f_max_) (Figure 9b) at the end of the elongation phase. At this position, the probe was retained for 60 s for the films to relax. While relaxing, the force values decreased exponentially with time. At the final stage of the relaxation phase, the force values reached a minimum value (f_min_) (Figure 9c). The SR profile of the composite films suggested that the addition of TG altered the firmness and elastic property of the films. Compared with the control film, the f_max_ value of all TG-containing films was decreased except in F4. However, the f_max_ value of F0 was statistically insignificant (*p* > 0.05) with all TG-containing films. This suggested that the firmness of the TG-containing films remained similar to F0, irrespective of variation in f_max_ values. Interestingly, the variation in the f_max_ values of F0 films was very high. Such a high variation suggested the formation of an inhomogeneous structure. The variation in f_max_ values of films correlates to the variations in the microarchitectures of the composite films. A dispersion of irregular and globular-shaped structures throughout the polymeric matrices of composite films may act as imperfections within the films, suggesting the variation in f_max_ value [67]. However, the f_max_ value was higher in F4, where the TG content was highest. The f_max_ value of F4 was statistically significant with the control film and other TG-containing films (*p* < 0.05). The increased firmness of F4 can be clarified as the cohesive forces between the PVA and TG molecules within the films were very strong, consequential forming of the dense composite film. The low swelling index of the highest TG-containing film (F4) can also be correlated with less water penetration of water due to the formation of dense hydrogel film. The residual force (f_min_) of the control film and TG-containing films found at the relaxation process end were the same as f_max_ values. The f_min_ value indicates the elasticity nature of the composite films. The f_min_ value was decreased initially in TG-containing films except in F4. The order of changes in f_min_ values in the composite films was seen as the same order of f_max_ values. The f_min_ value of F4 was highest but statistically insignificant with all films (*p* > 0.05). The films’ percentage relaxation (%SR) was computed from f_max_ and f_min_ using Equation (4). The %SR values of the sample have been used to gather information about the viscoelasticity nature of the product. The %SR is 100% for ideal fluids, while 0% is for an ideal elastic product. The %SR values for all films were about 80%. Interestingly, the range of relaxation was identical for all films, irrespective of the TG content within the films. This indicated that the increment of TG within the films did not substantially affect the viscoelastic property of the composite films. 

The normalized stress relaxation profile (Figure 9f,g) was fitted to the Weichert model to understand the composite films’ viscoelastic performance better. The parameters for the Weichert model were calculated using Equation (4) [45].

(3) $$\%\mathrm{SR}=\frac{f_{\max}-f_{\min}}{f_{\max}}\times 100$$

The films’ elasticity parameters (P_0_, P_1_, and P_2_) and viscosity parameters (τ1 and τ2) were tabulated in Table 3, and the fitted profiles are shown in Figure 9f. The correlation coefficient value between the investigational and model-fitted data in all the cases was greater than 0.99. The residual elastic energy towards the end of the relaxation process is represented by P_0_. Increasing TG content brought changes in the residual forces of the composite films. The P_0_ value of F_1_ was lower than the control film. With a subsequent increase in TG content within the film, the P_0_ value consequently increased in F2 and F3. The P_0_ value was decreased again in F_4_, where TG content was highest. However, the P_0_ value of all the films were similar (*p* > 0.05). The result suggested that the increased TG content did not significantly change the elastic component within the composite films. The time required to rearrange the polymeric structure under stress conditions is represented by the instantaneous relaxation time ‘τ1’. The result showed that the instantaneous relaxation time of the control film was statistically similar to all TG-containing films (*p* > 0.05). Among TG-containing films, τ1 value of F_1_ and F_4_ was increased compared to control films, whereas a decreased τ1 value was observed in F_2_ and F_3_. The τ1 value was lowest in F_3_. This indicated that the molecular rearrangement within F_3_ was fast. The higher τ1 value in F_4_ suggested the delay of molecular rearrangement owing to the dense composition. The τ1 value of F3 was statistically significant with F_1_ and F_4_ (*p* < 0.05). The longer relaxation time ‘τ2’ gives the information about the breakage of polymer chain interaction upon applying stress. The results indicated that the τ2 value of the control film, F_1_, F2, and F3, were similar (*p* > 0.05). The τ2 values of F2 and F4 were also identical (*p* > 0.05); however, the τ2 values of F4 were significantly higher than F0, F1, and F3 (*p* < 0.05). The higher long-duration relaxation time of F_2_ and F_4_ indicated the increased chances of polymer network breaking down under long-term stress conditions.

Furthermore, the stress relaxation profile, once normalized, was then fitted to Peleg’s model. The constants k_1_ and k_2_ were determined using Equation (5) [68]. The constant ‘k_1_’ indicates decay rate, and the other constant ‘k_2_’ display relaxation extent. The k_1_ value of the control film was statistically similar to F1, F2, and F3 (*p* > 0.05), and the k_1_ value of F3 was significantly lower than F0 and F4 (*p* < 0.05). The k_1_ value of F4 was significantly higher than F0 and F3 (*p* < 0.05). The highest k_1_ value of F4 can be explained by the early breakdown of the polymer network under applied stress. The extent of relaxation (k_2_) of all the formulations was similar (*p* > 0.05).

(4) $$P\left(t\right)=P_{0}+P_{1}\times e^{\frac{-\tau }{\tau 2}}+P_{2}\times e^{\frac{-\tau }{\tau 2}}$$

P(t) represents the variation in force concerning time; P_0_, the residual force at the termination of the relaxation phase, P_1_, and P_2_ are the constants for spring; τ1 and τ2 are the time constants (sec).

(5) $$\frac{\left(P_{0}\right)}{P_{0}}-P\left(t\right)=k_{1}+k_{2}\cdot t$$

where P_0_ represents the instant decaying force after loading, Pt represents decaying force at time ‘t’; and k_1_ and k_2_ denote the initial relaxation rate and the extent of relaxation, respectively.

### 2.7. Impedance Spectroscopy 

**Figure 10:**
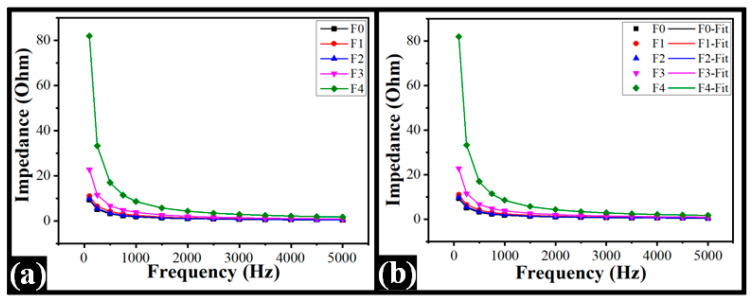
Electrical properties of the films: (**a**) Impedance profile and (**b**) model fitting RQ(Q) of the impedance profile.

The impedance profiles of the composite films were determined to understand the electrical properties of the films. The analysis was conceded in the frequencies ranging from 100 Hz to 5 kHz (Figure 10). The impedance profile of the pharmaceutical formulations has been placed as one of the important tools to predict the drug release profile from the formulations [69]. Additionally, the electrical properties of the formulations have a great role in iontophoretic drug delivery systems [70]. In general, the impedance of TG-containing films increased with the increase in TG content within the films, the exception being F2. The films with the highest TG content (F4) exhibited the maximum impedance, followed by F3, F1, F2, and F0, respectively. Such an observation can be explained by the increased quantum of phase-separated components in higher TG content films. The results showed decreased electrical impedance with an increase in frequency. The trend of the impedance values constantly persisted at higher frequencies. The capacitive-dominant electrical circuits typically exhibit this impedance profile [71].

The normalized impedance profiles were fitted to the RQ(Q) electrical model to perform a detailed examination of the electrical response of the synthesized films. The model parameters were calculated using Equation (5) [72]. Figure 10b demonstrated the fitted graph, and the model fitting parameters were tabulated in Table 4. The resistive component (R) values of the TG-containing films were higher than the control film. The F1 and F2 showed similar values. After that, the R-value was improved with the surge in TG content within the films. F4 showed the highest R-value. The changes in the capacitive component of the films caused the variation in the impedance profile. The constant phase element (Q) denotes a generalized capacitive element. It is commonly used in electrical modeling, where the capacitive component is anticipated to behave as a non-ideal capacitor. The capacitive component ‘Q’ of the control film was similar to TG-containing films F1 and F2. The Q value was consequently decreased following TG increment within F3 and F4, respectively. The capacitive component of F4 was the lowest, whereas TG content was the highest. The capacitive component of the sample-electrode interface (Q_1_) was similar for all films. The inhomogeneous constant associated with the “Q”, is known as the ‘n’ value. The value of n varies from 0 to 1. The ideal capacitance of the Q is indicated by the value of n equal to 1 [73]. The results for n values of all films were found in the range of 0.81 to 0.99. This suggested that the film capacitance was idealistic. The inhomogeneous constant (n1) is associated with Q_1_, and the value was 1.0 for all films. The constant for correlation between experimental and fitted data was observed as a good fit (R2 > 0.99).

(6) $$Z_{\mathrm{eq}}=\frac{R}{1+{\left(\mathrm{jw}\right)}^{n}Q\;R}+\frac{1}{{\left(\mathrm{jw}\right)}^{n\;1}Q_{1}}$$

Z_eq_ stands for equivalent impedance; R represents the resistance (U); Q and Q_1_ are the capacitance components; and n and n1 are the constants.

### 2.8. In Vitro Drug Release Study

**Figure 11:**
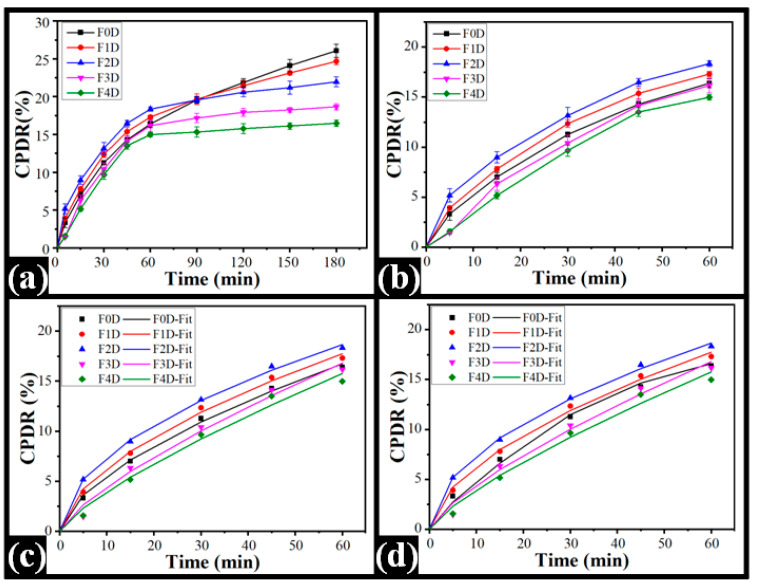
In vitro drug release from CPH-loaded composite films: (**a**) Profiles of %CPDR, (**b**) 60 min %CPDR profiles, (**c**) CPDR profiles fitting to KP model, and (**d**) PS model fitting of the CPDR profile.

Drug release is the transport of drug molecules from the carrier (polymeric or non-polymeric) matrix to the releasing medium. The carrier types and their concentrations within the delivery system affect the drug release. Figure 11a depicts the cumulative percentage drug release (CPDR) profiles obtained from the prepared films. The release of the drug was evaluated for 180 min. It was observed that the control film (F0) had the highest CPDR (26.07 ± 0.87%) compared with TG-containing films. The CPDR of the TG-included films was found in the order of F1D (24.68 ± 0.46%) > F2D (21.96 ± 0.66%) > F3D (18.47 ± 0.39%) > F4D (16.48 ± 0.41%). The CPDR from the composite films decreased with the increase in TG content. The impedance study further clarified such observation, where impedance values increased with increased TG content within the films. This suggests that the enhanced impedance has altered the hydrogel matrix’s ionic conductivity, decreasing the CPDR. The amount of drug released from PVA-TG films was lower than the control (pure PVA) film. In the case of composite films, CPDR decreased with the increase in TG concentration. However, the release of drugs from all the films was rapid in the first 60 min of the study. Thereafter, the CPDR of all TG-containing films decreased with the increase in TG content. The CPDR values of F1D and F2D were higher than the control film in the first 60 min of the diffusion study. Higher CPDR values for an initial 60 min can be reasoned to the presence of the drug on the surface of the films. Thereafter, the drug release was controlled according to the TG content within the films. A similar observation was observed in [71], where a drop in Moxifloxacin HCl release was observed when the TG content in the hydrogels was increased. 

The mechanism of drug diffusion from the films was studied for the rapid drug release in the initial 60 min of the study and was fitted to the Korsmeyer–Peppas model (KP model). The diffusion coefficient ‘K’ and the release exponent ‘n’ in the KP model were calculated using Equation (6), and the results are tabulated in Table 4. An increased K value was observed on TG increment within the film F1D and F2D compared to the control film. The K value of F2D was the highest (*p* < 0.05) among all films. Further increase in TG within the films decreased the K value in both F3D and F4D. This suggested the fact that the rate of diffusion of the drug was composition dependent. The diffusion rate of the drug from the polymeric composite film was controlled by the viscosity-building properties of TG [74]. The K value of F1D was similar to the K value of F3D and F4D (*p* > 0.05). The diffusional exponent (n) further explained the release mechanism of the drug from a polymeric matrix. The drug release mechanism may be Fickian transport (0.45 ≤ n), anomalous (non-Fickian) transport (0.45 ≤ n < 0.89), and/or super case-II transport (n > 0.89) [75]. The n values of the control film and all TG-containing films were greater than 0.45. This suggested that the drug release from all formulations followed anomalous transport. Thus, the KP model explains the drug release from the polymeric matrices is composition dependent and follows anomalous diffusion. Furthermore, the CPDR profile was fitted to the Peppas–Shalin model (PS model) to examine the influence of both Fickian diffusion and relaxation of polymers in releasing Ciprofloxacin HCl from the composite polymeric matrices. The Fickian diffusion constant (k_d_) and the polymer relaxation constant (k_r_) were calculated using Equation (7) and tabulated in Table 5. The results of the PS model parameter designated that the polymer relaxation influenced the mechanism of diffusion from the composite films.

(7) $$F=K\cdot t^{n}$$

where F is the solute fraction released, K is the release rate constant, t is the sampling time, and n signifies the diffusion exponent.

(8) $$F=k_{d}\cdot t^{m}+k_{r}\cdot t^{2m}$$

where k_d_, k_r_, and m represent the Fickian diffusion constant, polymer relaxation constant (case II), and the diffusion exponent, respectively.

### 2.9. Antimicrobial Study

**Figure 12:**
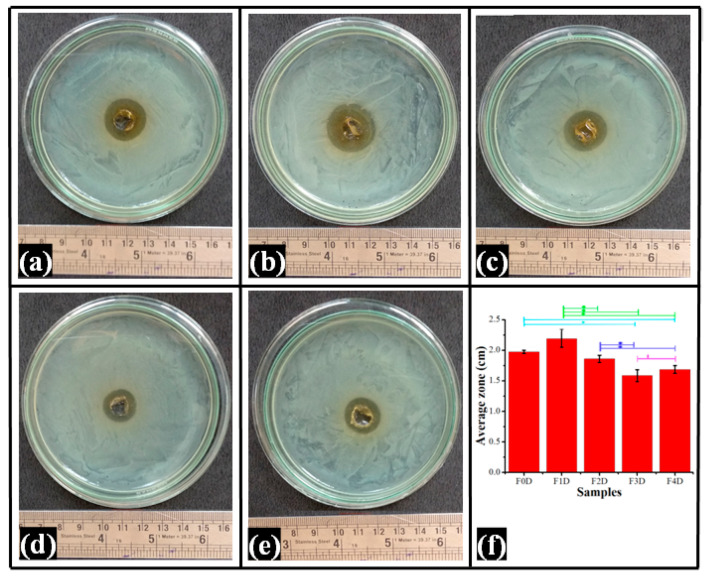
(**a**–**e**): Pictures displaying the zone of inhibition. (**f**) Quantification of antibacterial activity of CPH-loaded films. The values in the graph are denoted as the mean of the triplicate ± standard deviation. The different values (*p* < 0.05) are represented with symbol “*”.

Ciprofloxacin (1-cyclopropyl-6-fluoro-4-oxo-7-(piperazine-1-yl)-1,4-dihydroquinoline-3-carboxylic acid hydrate, CPH) is the most effective fluoroquinolone antibiotic against Gram-negative bacilli bacteria such as Escherichia coli, Salmonella spp., Shigella spp., and diplococci Neisseria spp. [45,74]. It was also found to be effective against some Gram-positive strains [76]. Their key mechanism of action includes the inhibition of bacterial DNA gyrase, which prevents bacterial growth and proliferation. The drug-loaded films were studied for their antibacterial effectiveness against E. coli. The clear zone of inhibition of the drug-loaded films indicated their effective antibacterial activity against E. coli. The difference in the ZOIs indicates that drugs are active inside the films and are also capable of being released from the matrix. The ZOI of the composite films depends on the swelling of the films in the culture media and the partitioning of the dissolved drug to the culture media. The result of ZOI confirms the antibacterial activity of all the films. The slower diffusion of the drug from the films containing higher TG content showed smaller ZOIs [66]. The diameter of ZOI for F0D was 1.974 ± 0.025 cm. Except for F1D, the ZOIs of TG-containing films were found to decrease as the TG content increased. The F1D ZOI diameter was the highest of all films but statistically insignificant regarding the control film and F2D (Figure 12f). Similarly, the ZOI diameters of F3D and F4D were similar (*p* > 0.05). The antibacterial activity of CPH-loaded PVA films synthesized by Lian et al. (2021) [77] and David et al. (2021) [78] showed results similar to the obtained results.

## 3. Materials and Methods

PVA was procured from Loba Chemie Pvt. Ltd.,Mumbai, Maharashtra, India. Glutaraldehyde (GTA) and hydrochloric acid were procured from Molychem, Mumbai, Maharashtra, India. Tamarind gum (molecular weight 6.97 × 105 g/mol) was obtained from Maruti Hydrocolloids, Ahemdabad, India. Alkem Laboratories Limited, Sikkim, India, provided a free ciprofloxacin HCl (CPH) sample. Nutrient broth and nutrient agar used for the antimicrobial study were obtained from Hi-Media Pvt. Ltd., Mumbai, Maharashtra, India. Double-distilled water was used throughout the study.

### 3.1. Preparation of Films

At first, 10% (*w*/*w*) PVA solution completely dissolved 10.0 g of PVA in 90.0 g of hot water (70 °C). TG suspensions (1.0%, 2.5%, 5.0 %, and 10.0%) were prepared by dispersing TG powder in water and subsequent homogenization for 20 min using an overhead stirrer (800 rpm). The suspensions were prepared at room temperature (25 °C). The PVA solution and the TG suspension were mixed in the ratio of 9:1 and then homogenized through an overhead stirrer (800 rpm, 15 min). The mixtures were then diluted with 20 mL water. In total, 2 mL of crosslinking reagent (5 mL GTA, 5 mL water, and 0.5 mL HCl) was added to the diluted mixture and homogenized for 1 min. The mixture was then degassed by sonicating it for 30 min in a bath sonicator. In total, 10 g of the final suspension was poured into Petri plates and incubated at 40 °C for 24 h. Following the abovementioned approach, the liquid suspensions were converted into thin films. 

The film without TG suspension was prepared using 20.0 g of the PVA solution. The PVA film was used as the negative control. Drug-loaded films were made by adding 100 mg of ciprofloxacin HCl to the PVA solution and TG suspension mixture before homogenization. The rest of the steps remained the same. Table 6 displays the composition of the prepared films.

### 3.2. Microscopy

The films (1 cm × 1 cm) were examined under the microscope. The microstructure of the prepared films was examined under a brightfield microscope (model: DM750, Leica, Wetzlar Germany).

### 3.3. Swelling Study

The swelling property of the films was determined using double-distilled water at room temperature. Two mL of double-distilled water was poured onto a watch glass containing pre-weighted square films (20 mm × 20 mm) and allowed to swell for 2 min. The water was carefully removed, and the film’s weight was recorded. The same volume of distilled water was then substituted, and the procedure was continuously monitored until the swollen weight of the films remained constant. The weight was measured at an interval of 2 min for the first 10 min, followed by 5 min for another 30 min. Equation (8) was used to calculate the swelling index [79].

(9) $$\%Swelling=\frac{W_{t}-W_{0}}{W_{0}}\times 100$$

*W_t_* is the weight at the time t of swollen film and *W*_0_ is the film’s weight before swelling.

### 3.4. Transparency Study

The clarity and transparency of the films were observed visually by reading the numbers on a standard measuring ruler through the films. The evidence of clarity was recorded by taking the digital photograph against the measuring ruler.

Furthermore, a UV–vis spectrometer was used to measure the transparency of the prepared films (UV-1700, Shimadzu Corporation, Duisburg, Germany). The spectrometer was corrected for the baseline correction using the PVA film as a control. Rectangular shape films were cut and inserted into the spectrophotometer’s cuvette holder. The percent transmittance (%T) was then measured by scanning the 280–900 nm wavelength range films. 

### 3.5. Loss on Drying

The percentage of moisture loss during drying and loss on drying (LOD) was determined using the moisture analyzer (Dolphin Instruments, Mumbai, India). For this purpose, the LOD of the films was determined by putting the films (1 g) on the moisture analyzer pan. Subsequently, the films were heated at 60 °C until the weight of the film was constant. The procedure was repeated in triplicate.

### 3.6. FTIR Analysis

FTIR spectroscopy was carried out to investigate the molecular interactions between the films’ components. Infrared spectra of the films were obtained in the Attenuated Total Reflectance (ATR) mode. An ATR-FTIR (AlphaE ATR-FTIR, Bruker, Billerica, MA, USA) spectrometer was used for the analysis. The prepared films were placed on the ATR crystal. The spectra were scanned over a wavenumber range of 4000 cm^−1^ to 500 cm^−1^.

### 3.7. Impedance Analysis

The electrical properties of the films were investigated by measuring their impedance. The films were clamped between the stainless steel electrodes (diameter: 1 cm). The impedance values were measured in the frequency range, i.e., 100 Hz to 5 kHz, utilizing the NI-ELVIS-II system (National Instruments, Austin, TX, USA).

### 3.8. Mechanical Studies

The mechanical properties of the prepared films were investigated by studying their stress relaxation profiles and tensile strength. The analysis was performed using a mechanical tester (TA HDplus, Stable Micro Systems, Haskmere, England). Rectangular pieces of the films (50 mm × 10 mm) were cut and held in sample holders such that the sample length of the window of the sample holder was 40 mm. Then, the sample holder was attached to the tensile grips, followed by tearing the sample holder’s sides. Thereafter, the films were stretched for a distance of 5 mm at a speed of 1 mm/s. The probe was allowed to stay at the said position for 60 s to allow the relaxation process. The continuous change in the force values was recorded during the hold time.

### 3.9. In Vitro Drug Release

The in vitro drug release of CPH from the films was measured using Franz’s diffusion cell. The releasing medium, i.e., 12 mL of phosphate-buffered solution (PBS, pH, 7.2), was kept in the receiver compartment. The dialysis membrane (MW cut-off: 60 KDa) was placed between the donor and receiver compartments after being hydrated in PBS for 12 h. The cell’s diffusion area was 0.64 cm^2^. After placing a section of the film (9 mm × 9 mm) over the dialysis membrane, the receptor fluid was continuously stirred at 37 °C (300 rpm). In total, 1 mL of receptor fluid was withdrawn from the receptor compartment at regular intervals. Fresh PBS was used to replace the sampled receptor fluid. After appropriate dilution, the receptor fluid was examined for drug content via a UV–vis spectrophotometer (UV-1700, Shimadzu Corporation, Duisburg, Germany) at 277 nm.

### 3.10. Antimicrobial Study

The antimicrobial activity against Gram-negative bacteria (Escherichia coli) of drug-loaded films was analyzed. In total, 100 µL of the microbial cell suspensions (109 CFU/mL) were evenly spread on agar plates and a borer was used to make a 10 mm hole in the center. The films containing CPH were punched into discs (10 mm in diameter) and placed in the well. As a negative control, blank films (no drug) were used. The plates were incubated at 37 °C for 12 h before the inhibition zones were observed.

### 3.11. Statistical Studies

All the studies were performed in triplicate, representing the data as mean ± S.D. The analysis of significance (*p* < 0.05) was carried out through the Student’s *t*-test.

## 4. Conclusions

This study substantiates the influence of TG on PVA-based composite films and proves its potential to be used as an additive in pharmaceutical formulations. TG enhanced the phase separation of PVA from the composite films and facilitated an interconnected network of phase-separated PVA-TG aggregates. The addition of TG seems to have improved the optical and swelling properties of the films. However, TG has no significant role in influencing either the firmness or the viscoelastic properties of films. On the other hand, Peleg’s analysis of stress relaxation profiles indicated that the films become brittle (based on the long-duration relaxation time) with the increase in TG concentration. Fitting the in vitro CFX release data in KP and PS models has indicated non-Fickian diffusion of CFX from the films. The decrease in CFX release with the increase in TG was attributed to the corresponding reduction in impedance and altered ionic conductivity in films. Based on the extensive characterization and drug release kinetics studies, TG must be able to fine-tune the physicochemical properties of PVA-TG composite films toward a controlled release system.

## Figures and Tables

**Table 1:** Swelling parameters of the composite films.

Study	Model	Parameter	Formulations				
			F0	F1	F2	F3	F4
% swelling	Korsmeyer -Peppas	k	54.64 ± 8.05 ^a^	158.99 ± 10.18 ^bd^	176.03 ± 6.43 ^bc^	161.59 ± 4.95 ^d^	75.02 ± 4.60 ^e^
		n	0.42 ± 0.07 ^abcd^	0.38 ± 0.02 ^a^	0.53 ± 0.01 ^b^	0.54 ± 0.01 ^bc^	0.32 ± 0.02 ^d^
		R^2^	0.99 ± 0.00	0.99 ± 0.00	0.99 ± 0.00	1.00 ± 0.00	1.00 ± 0.00
	Peppas -Shalin	kd	0.00 ± 0.00	0.00 ± 0.00	0.00 ± 0.00	0.00 ± 0.00	0.00 ± 0.00
		kr	54.64 ± 8.05 ^a^	158.99 ± 10.18 ^bd^	176.03 ± 6.43 ^bc^	161.59 ± 4.95 ^d^	75.02 ± 4.60 ^e^
		m	0.21 ± 0.04 ^abcd^	0.19 ± 0.01 ^a^	0.26 ± 0.01 ^b^	0.27 ± 0.00 ^bc^	0.16 ± 0.01 ^d^
		k_d_/k_r_	0.00 ± 0.00	0.00 ± 0.00	0.00 ± 0.00	0.00 ± 0.00	0.00 ± 0.00
		R^2^	0.99 ± 0.00	0.99 ± 0.00	0.99 ± 0.00	1.00 ± 0.00	1.00 ± 0.00

Superscripts with different alphabets in the same row represent significantly (*p* < 0.05) different values.

**Table 2:** Important peaks obtained from FTIR spectra.

Frequency Range (cm^−1^)	Functional Group	References
3298	Stretching vibration (-OH)	[56]
2933.1	Stretching of the alkyl (C-H)	[57]
1721.1	Stretching vibration carbonyl (C=O)	[58,59]
1655.1	stretching vibration (C=C)	[60]
1564.4,1424.3	Bending vibration (-CH2)	[60]
1370.7, 1327.4	Bending vibration of -CH3)	[61,62,63]
1249.1	C-O-C	[60]
1086.2, 1032.6	Stretching(C-O)	[64]
958.4	Bending vibration of Alkyl (C-H)	[64]
836.8	Rocking vibration of Alkyl (C-H)	[64]

**Table 3:** Mechanical parameters.

Model	Parameter	F0	F1	F2	F3	F4
	ƒ max	1517.04 ± 95.38	1078.27 ± 152.33	1439.90 ± 155.14	1152.66 ± 247.09	2373.57 ± 140.98
	ƒ min	286.71 ± 31.61	197.07 ± 34.72	276.38 ± 20.92	230.00 ± 40.35	351.46 ± 80.35
	%SR	81.14 ± 0.93	81.76 ± 0.82	80.72 ± 1.21	79.91 ± 1.21	85.12 ± 5.39
**Weichert model**	P_0_	0.19 ± 0.01 ^a^	0.19 ± 0.01 ^b^	0.20 ± 0.01 ^c^	0.21 ± 0.02 ^d^	0.15± 0.05 ^e^
	P_1_	0.52 ± 0.01 ^a^	0.51 ± 0.01 ^b^	0.52± 0.01 ^c^	0.52 ± 0.01 ^d^	0.53 ± 0.03 ^e^
	τ1	0.82 ± 0.04 ^a^	0.83 ± 0.01 ^b^	0.79 ± 0.03 ^c^	0.72 ± 0.02 ^abcd^	0.83± 0.03 ^d^
	P_2_	0.27± 0.01 ^a^	0.29 ± 0.01 ^b^	0.27 ± 0.00 ^c^	0.268 ± 0.00 ^d^	0.312 ± 0.03 ^e^
	τ2	12.76± 0.66 ^a^	12.64 ± 0.35 ^b^	12.93 ± 2.11 ^c^	12.33 ± 0.73 ^d^	14.60 ± 0.50 ^ab^
	R^2^	1.00 ± 0.00	1.00 ± 0.00	1.00 ± 0.00	1.00 ± 0.00	1.00 ± 0.00

Superscripts with different alphabets in the same row represent significantly (*p* < 0.05) different values.

**Table 4:** RQ(Q) parameters of the impedance obtained through the model fitting.

Parameters	Formulations				
	F0	F1	F2	F3	F4
R (Q)	17.87	19.51	19.07	61.19	518.06
Q (µF)	355.20	334.56	372.78	154.34	20.09
Q_1_ (µF)	2.67	2.67	2.67	2.67	2.67
n	0.84	0.81	0.82	0.85	0.99
n_1_	1.00	1.00	1.00	1.00	1.00
R^2^	1.00	1.00	1.00	1.00	1.00

**Table 5:** In vitro drug release parameters.

Study	Model	Parameter	Formulations				
			F0D	F1D	F2D	F3D	F4D
Diffusion	Korsemeyer –Peppas	k	1.37 ± 0.29	1.67 ± 0.05	2.29 ± 0.43	0.80 ± 0.13	0.67 ± 0.11
		n	0.62 ± 0.06	0.58 ± 0.01	0.51 ± 0.04	0.75 ± 0.03	0.77 ± 0.04
		R^2^	1.00 ± 0.00	1.00 ± 0.00	1.00 ± 0.00	0.99 ± 0.00	0.99 ± 0.00
	Peppas –Shalin	k_d_	0.00 ± 0.00	0.00 ± 0.00	0.00 ± 0.00	0.00 ± 0.00	0.00 ± 0.00
		k_r_	1.36 ± 0.29	1.67 ± 0.05	2.01 ± 0.21	0.80 ± 0.13	0.67 ± 0.11
		m	0.31 ± 0.03	0.29 ± 0.00	0.27 ± 0.01	0.37 ± 0.01	0.39 ± 0.02
		k_d_/k_r_	0.00 ± 0.00	0.00 ± 0.00	0.00 ± 0.00	0.00 ± 0.00	0.00 ± 0.00
		R^2^	1.00 ± 0.00	1.00 ± 0.00	1.00 ± 0.00	0.99 ± 0.00	0.99 ± 0.00

**Table 6:** Formulation of the PVA-TG films.

Composition	Formulations									
	F0	F1	F2	F3	F4	F0D	F1D	F2D	F3D	F4D
PVA solution (g)	20	18	18	18	18	20	18	18	18	18
Tamarind gum suspension (g)	--	2 (1% *w*/*v*)	2 (2.5% *w*/*v*)	5 (5% *w*/*v*)	2 (10% *w*/*v*)	--	2 (1% *w*/*v*)	2 (2.5% *w*/*v*)	5 (5% *w*/*v*)	2 (10% *w*/*v*)
Ciprofloxacin HCl (g)	--	--	--	--	--	0.1	0.1	0.1	0.1	0.1
Crosslinking solutions (mL)	2	2	2	2	2	2	2	2	2	2
Distilled water (g)	20	20	20	20	20	20	20	20	20	20

## Data Availability

The data presented in this study are available on request from the corresponding author.
